# Flexible regulation of DNA displacement reaction through nucleic acid-recognition enzyme and its application in keypad lock system and biosensing

**DOI:** 10.1038/s41598-017-10459-y

**Published:** 2017-08-30

**Authors:** Chao Li, Liu Shi, Yaqin Tao, Xiaoxia Mao, Yang Xiang, Genxi Li

**Affiliations:** 1 State Key Laboratory of Pharmaceutical Biotechnology and Collaborative Innovation Center of Chemistry for Life Sciences, Department of Biochemistry, Nanjing University, Nanjing, 210093 P.R. China; 2 Center for Molecular Recognition and Biosensing, School of Life Sciences, Shanghai University, Shanghai, 200444 P.R. China

## Abstract

Toehold-mediated DNA strand displacement reaction (SDR) plays pivotal roles for the construction of diverse dynamic DNA nanodevices. To date, many elements have been introduced into SDR system to achieve controllable activation and fine regulation. However, as the most relevant stimuli for nucleic acid involved reaction, nucleic acid-recognizing enzymes (NAEs) have received nearly no attention so far despite SDR often takes place in NAEs-enriched environment (i.e., biological fluids). Herein, we report a set of NAEs-controlled SDR strategies, which take full advantage of NAEs’ properties. In this study, three different kinds of enzymes belonging to several classes (i.e., exonuclease, endonuclease and polymerase) have been used to activate or inhibit SDR, and more importantly, some mechanisms behind these strategies on how NAEs affect SDR have also been revealed. The exploration to use NAEs as possible cues to operate SDR will expand the available toolbox to build novel stimuli-fueled DNA nanodevices and could open the door to many applications including enzyme-triggered biocomputing and biosensing.

## Introduction

In DNA nanotechnology, toehold-mediated DNA strand displacement reaction (SDR) is a powerful tool to construct many dynamic devices such as nanomachines^1–3^, sensors^4, 5^, logic gates^6, 7^, transformable structures^8–10^ and catalytic amplifiers^11^. In general, the key feature of SDR is DNA strand-exchange reaction in which a prehybridized strand from a DNA duplex is displaced by an invading DNA strand. The introduction of toehold significantly enhances the migration rate of invading strand toward original duplex to over 10^6^-fold^12^, laying a foundation for dynamic DNA nanotechnology.

Originally, SDR is roughly manipulated by varying the toehold binding strength. Later, in order to enrich the activatable toolbox of SDR, different kinds of improved SDRs which are based on the toehold design, including toehold exchange^13^, remote toehold^14^, associative toehold^15^, and allosteric toehold^16^, have been successively developed. Moreover, considering the above strategies only concentrate on pure DNA systems, many efforts have also been made to realize SDR that is responsive to other stimuli instead of oligonucleotides. For example, aptamers have been integrated into toehold sequence, making the SDR responds to adenosine triphosphate (ATP)^17, 18^. Protein enzymes such as glutathione transferase and urease that can induce the pH change of solution have been used to trigger the activation of SDR^19^. Environmental factors such as light, pH, and metal ions have also been employed as stimulating elements for SDR through photocleavable oligonucleotides and DNA tetraplexe structures^20, 21^. For example, Ricci group developed a set of pH-responsive DNA devices in which the SDR can be finely triggered and controlled by using the parallel Hoogsteen (T, C)-motif in triplex DNA^22^. Altogether, the exploration of novel responsive elements for SDR has two fundamental significances: (1) it helps in improving the programmability of this reaction since in the presence of invading strand the process can’t be further controlled; (2) it expands potential utilization of this reaction in biomedical areas such as biosensor and targeted drug delivery.

Nucleic acid-related enzymes (NAEs), including nuclease, polymerase, and nicking enzymes, etc., have been widely used in molecular biology and many important techniques such as polymerase chain reaction (PCR) and gene recombination technology. Several groups have also made use of NAEs to dynamically control DNA nanodevices^23, 24^. Nevertheless, we note that there has been no report on controlling SDR through NAEs so far^25^. Considering SDR has been involved in the real biological environment (i.e., translation regulator)^26, 27^, the interaction between SDR’s elements and cellular NAEs is unavoidable. So, it is vital to study NAEs-controlled SDRs, which not only provides novel regulation approaches to the current strand displacement toolbox, but also helps in understanding the influence of NAEs on SDR and SDR-based DNA nanodevices. Motivated by the above arguments, we have conducted a series of NAEs-based studies for flexible SDR control and have further made use of these strategies to realize keypad lock systems and biosensing.

## Results and Discussion

### Nuclease-regulated SDR

**Figure 1:**
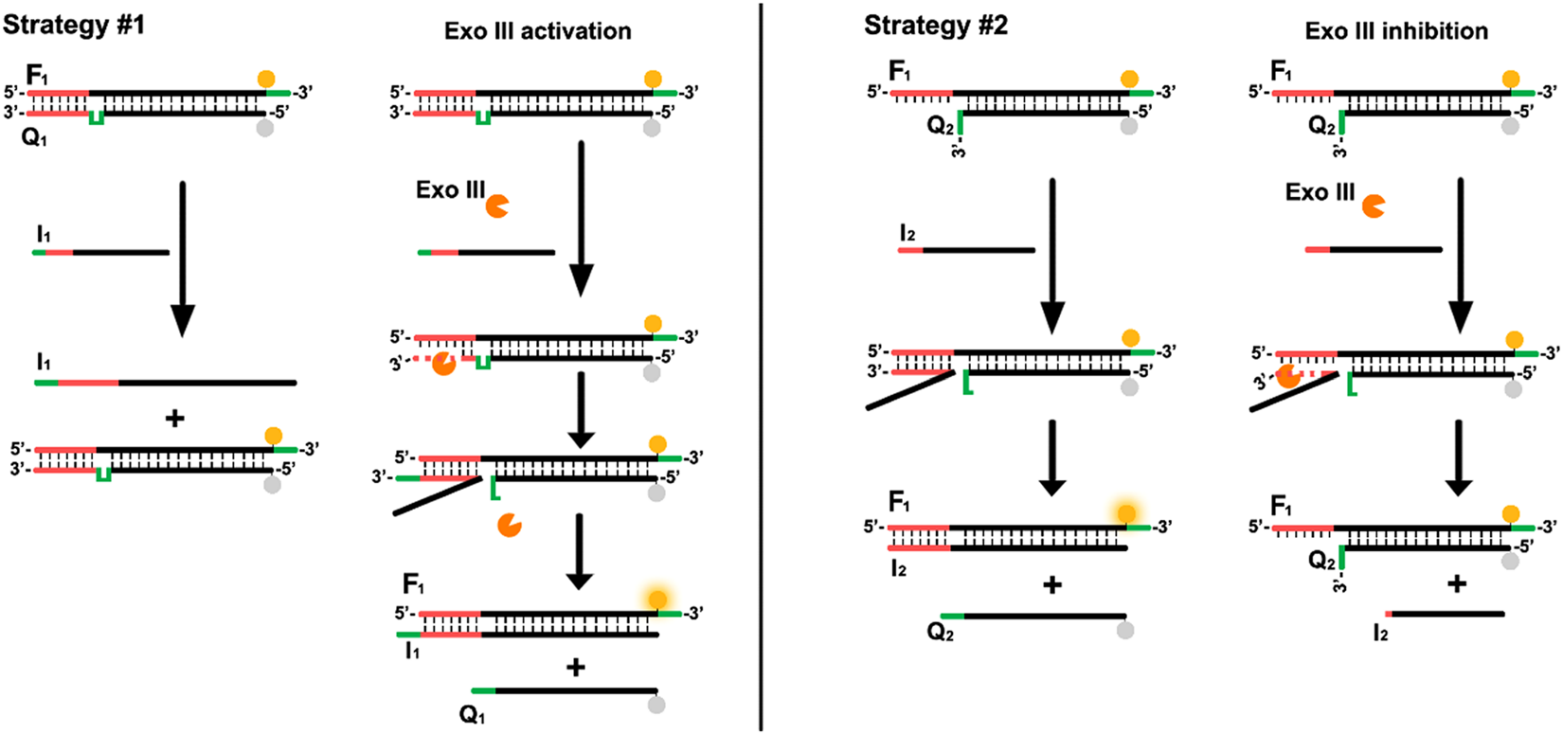
General scheme of the Exo III-controlled, toehold-based DNA strand displacement strategies.

Generally, there are two primary classifications of nuclease, namely exonucleases and endonucleases. However, some nucleases such as DNAase I and S1 nuclease indiscriminately cleave single-stranded DNA (ssDNA) or double-stranded DNA (dsDNA) without sequence-selectivity, making them impossible to regulate sequence-dependent SDR. So, we choose exonuclease III (Exo III), a classic exonuclease that selectively digests the 3′-end of strands in duplex DNAs, to realize SDR control. Figure 1 schematically outlines two principles of the Exo III-controlled SDR, which employ Exo III as an activator (strategy #1) or an inhibitor (strategy #2), respectively. In the first strategy, two ssDNA strands, F_1_ (fluorophore unit modified strand) and Q_1_ (quencher modified strand), both have domains complementary to each other, and the two strands also contain additional unpaired domains (4 nucleotides, green) in between. Strand F_1_ has an overhang at its 3′-end that can efficiently block the unfavorable cleavage of Exo III after hybridization of F_1_ and Q_1_, and the same overhang also appears in the 3′-end of strand I_1_ (invading strand). After the formation of F_1_Q_1_ duplex, the unpaired domain of Q_1_ forms a loop close to the toehold (red) that is hidden in the duplex, resulting in an extremely slow strand displacement between strand I_1_ and F_1_Q_1_ duplex. In the presence of Exo III, enzyme cleave happens at the 3′-end of Q_1_ until Exo III meets the bulge-loop structure without destroying the whole strand Q_1_. As a result, the hidden toehold of the F_1_Q_1_ duplex can be exposed and hybridize with its complementary strand I_1_ to initiate the SDR. In the second strategy, since the toehold has been exposed directly, we expect that the Exo III could rapidly cleave from the 3′-end of strand I_2_ once the SDR starts, leading to the failure of DNA displacement.

**Figure 2:**
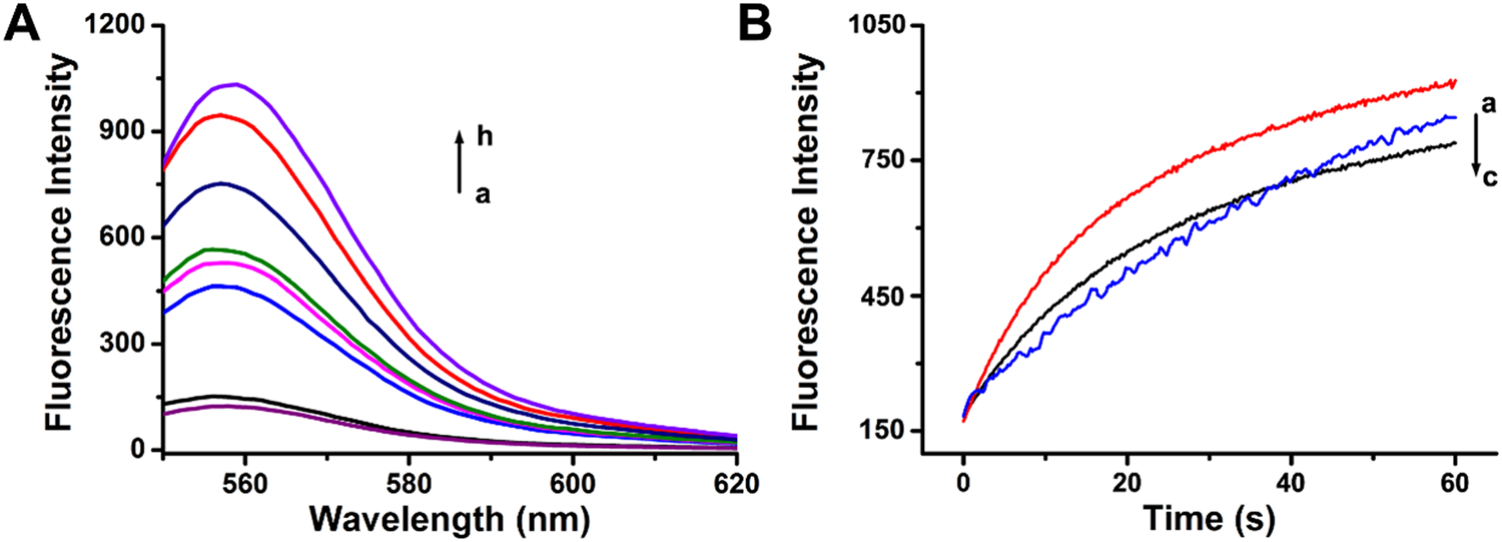
(**A**) Time-dependent fluorescence spectra changes upon subjecting the Exo III activation module to only invading strand I_1_ (a) and I_1_ and variable concentrations of Exo III: (b) 0, (c) 1, (d) 2, (e) 3, (f) 5, (g) 10 units, and (h) only strand F_1_, [F_1_] = [Q_1_] = [I_1_] = 50 nM. (**B**) Real-time fluorescence changes upon treatment of the Exo III inhibition module with I_2_ and variable concentrations of Exo III: (a) 0, (b) 5, and (c) 10 units, [F_1_] = [Q_2_] = [I_2_] = 50 nM.

Figure 2A depicts the results of strategy #1 using Exo III as an activator. When the F_1_Q_1_ duplex forms, the fluorophore HEX (λ_em_ = 536 nm and λ_ex_ = 556 nm) and the black-hole quencher (BHQ1) are in proximity to each other, so the fluorescence is quenched. As expected, the addition of strand I_1_ cannot recover fluorescence signal, demonstrating that no displacement occurs. Similarly, Exo III alone also fails to enhance any signal response. However, once in the presence of Exo III and strand I_1_, SDR can be activated in an enzyme-concentration-dependent manner, consistent with the formation of the energetically stabilized duplex F_1_I_1_. Upon treatment of enzyme (10 units) within 4 min, almost 92% strand Q_1_ has been displaced by strand I_1_, suggesting a high activation level. These results demonstrate that Exo III indeed acts as an activator and can regulate SDR by adjusting the concentration of Exo III molecules.

Figure 2B depicts the results of strategy #2 using Exo III as an inhibitor. Since the toehold (8 nucleotides) has already been exposed, the addition of strand I_2_ can significantly increase fluorescence signal. However, when Exo III has been introduced into the system, the fluorescence signal doesn’t reduce obviously even at the highest Exo III concentration (10 units), indicating that Exo III has weak ability to stop SDR. It is worthy to note that the source of signal recovery is not due to the Exo III-induced dissociation of F_1_ from the formed F_1_/I_2_ duplex, considering that the completely cleavage of I_2_ in the F_1_/I_2_ duplex requires ~8 min (Figure S1). Actually, SDR can be divided into two processes, which are toehold binding and subsequently branch migration. If Exo III has enough hydrolysis speed, the toehold of strand I_2_ will be timely destroyed before the branch migration begins. Nevertheless, these results suggest that SDR is so fast that Exo III doesn’t has enough time to completely cleave toehold to stop SDR, which may be attributed to the relatively low processivity of the enzyme. Although Exo III fails to act as an inhibitor, this phenomenon may be helpful to understand the DNA displacement behavior when the system appears Exo III that has been widely used in nucleic acid-related areas.

### Nicking enzyme-controlled SDR

**Figure 3:**
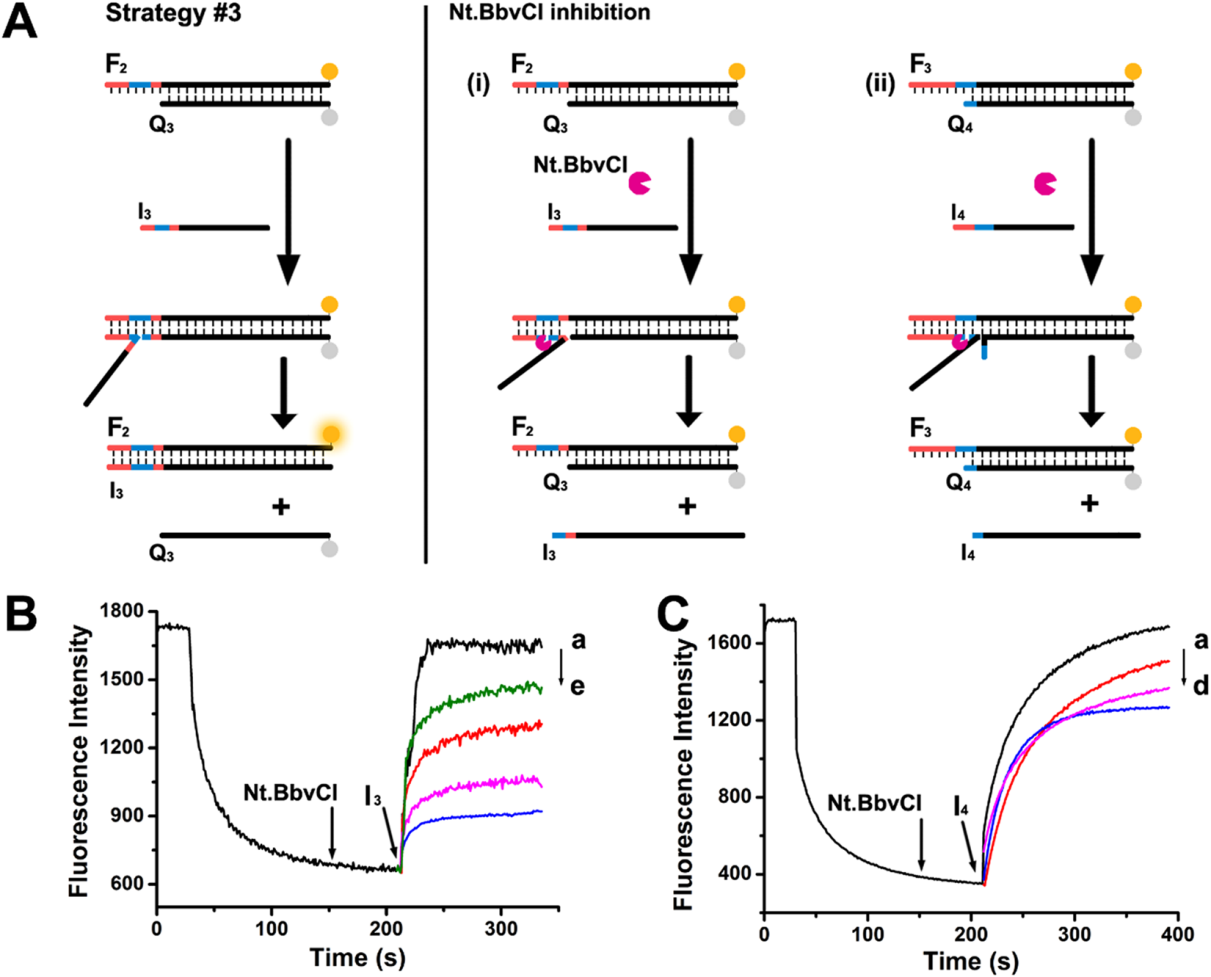
(**A**) General scheme of the Nt.BbvCl-inhibited, toehold-based DNA strand displacement strategies. (**B**) Real-time fluorescence changes upon treatment of the Nt.BbvCl-inhibition module i with variable concentrations of Nt.BbvCl: (a) 0, (b) 1, (c) 2, (d) 5, and (e) 10 units, [F_2_] = [Q_3_] = [I_3_] = 50 nM. (**C**) Real-time fluorescence changes upon treatment of the Nt.BbvCl-inhibition module ii with variable concentrations of Nt.BbvCl: (a) 0, (b) 1, (c) 5, and (d) 10 units, [F_3_] = [Q_4_] = [I_4_] = 50 nM.

Nicking enzyme (NEase) is a common endonuclease that recognizes specific nucleotide sequences in double-stranded DNA and cleaves only one of the two strands. Different from Exo III, NEases don’t cleave nucleotides one by one, while they can create a nick in the DNA duplex, which may be superior candidates for SDR regulation. Figure 3A schematically outlines two principles of the NEase-controlled SDR, which use NEase as an inhibitor. The recognition sequence of (blue) Nt.BbvCl is integrated into toehold, and we expect that Nt.BbvCl could rapidly cleave and shorten the toehold nucleotides in invading strand from seven to two, resulting in an oppressive SDR.

Figure 3B depicts the outcomes of strategy #3 using Nt.BbvCl as an inhibitor. Considering the property of NEase, the location of recognition sequence has two potential sites. One is entirely located in the toehold and the other is located between toehold and branch migration sequence (strategy #3i and ii). We have studied the displacement processes before and after injection of Nt.BbvCl through recording the real-time fluorescence change. For both strategies, Nt.BbvCl alone cannot induce any signal increase, while strand displacement proceeds with a fast kinetic upon invading strand I_3_/I_4_ addition (Fig. 3B and C, curve a). However, once in the presence of Nt.BbvCl, the strand displacement rates can be decelerated in a Nt.BbvCl concentration-dependent way. When Nt.BbvCl concentrations are increased from 1 to 10 units (Fig. 3B curve b–e, Fig. 3Cb–d), the rate constants can be regulated in the range from 8.69 × 10^5^ to 7.22 × 10^4^ M^−1^ s^−1^ (strategy #3i) and from 1.21 × 10^5^ to 9.94 × 10^4^ M^−1^ s^−1^ (strategy #3ii), respectively. These results are consistent with the Nt.BbvCl-stimulated SDR inhibition mechanism, and the strand displacement kinetics can be finely adjusted by varying the concentrations of Nt.BbvCl.

Obviously, Nt.BbvCl in strategy #3i has a much better inhibition effect compared with strategy #3ii. Considering the most difference between strategy #3i and ii is the location of enzyme’s recognition site, it indicates that toehold binding is the rate-determining step, so Nt.BbvCl has enough time to nick toehold; however, once the whole toehold completes hybridization and branch migration process proceeds, the displacement reaction rate greatly promotes, making Nt.BbvCl inefficiently prevents SDR. So, we conclude that the reaction rate order is r_branch migration_ > r_Nt.BbvCl_ > r_toehold binding_ > r _Exo III_.

### Polymerase enzyme-controlled SDR

**Figure 4:**
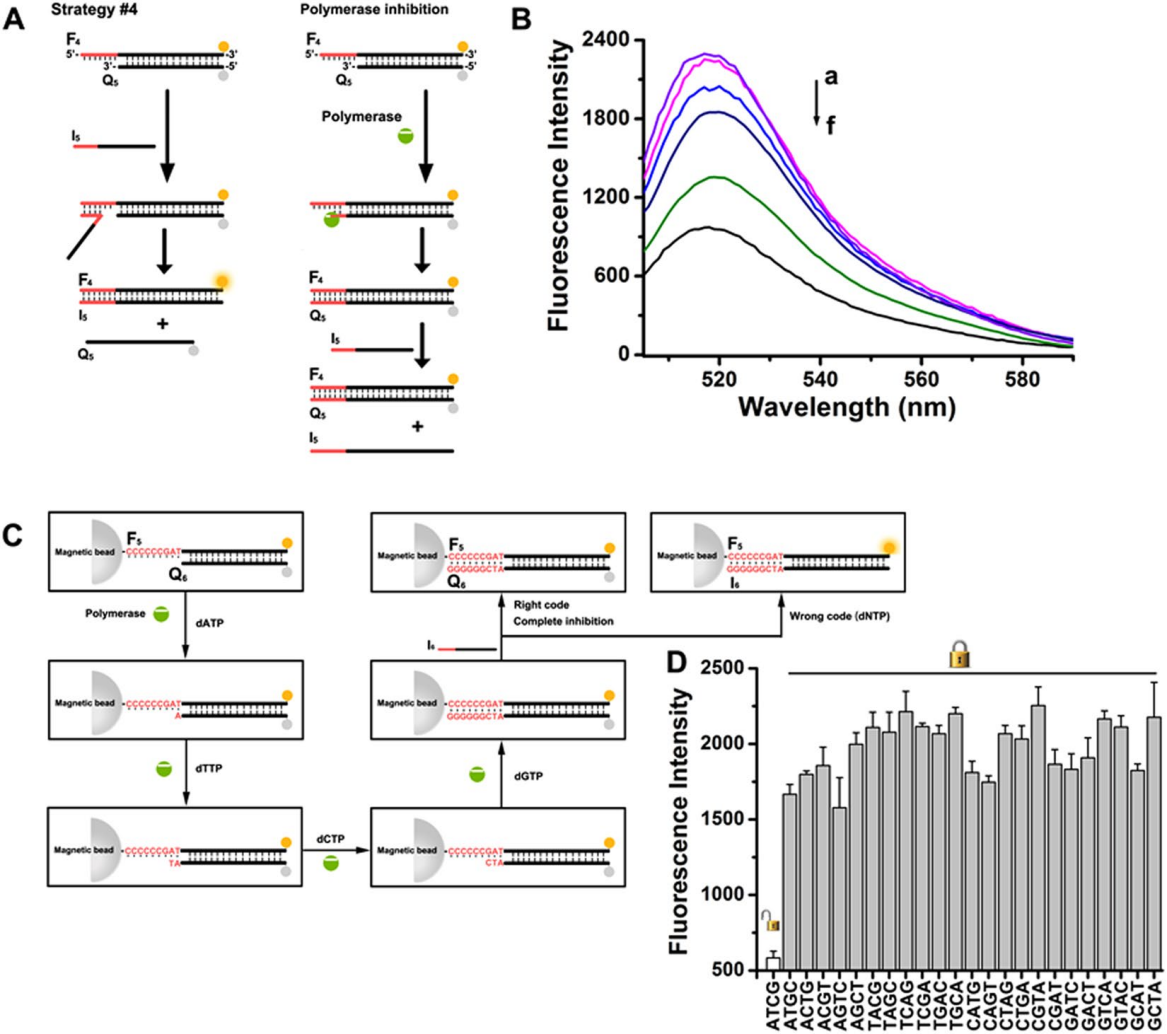
(**A**) General scheme of the polymerase-inhibited, toehold-based DNA strand displacement strategy. (**B**) Fluorescence spectra changes observed upon treatment of the sensing module with variable concentrations of polymerase: (a) 0, (b) 1 × 10^−5^, (c) 1 × 10^−4^, (d) 1 × 10^−3^, (e) 0.01, and (f) 0.1 units, [F_4_] = [Q_5_] = [I_6_] = 50 nM. (**C**) General scheme of the polymerase-based keypad lock system. (**D**) Fluorescence intensities of the system at 520 nm with respect to 24 kinds of input sequences.

Polymerase can add nucleotides from 3′-end of template strand with extremely high efficiency (1000 nucleotides/min). Thus, it is easily to achieve polymerase-inhibited SDR. As shown in Fig. 4A, strand F_4_ and Q_5_ are partially complementary to each other, and the extension of strand Q_5_’s 3′-end by polymerase can efficiently block toehold and suppress SDR. Figure 4B shows the results obtained from addition of different concentrations of polymerase to the reaction system. Of note, without any optimization, the SDR is responsive to polymerase as low as 1 × 10^−5^ units, which is much more sensitive than nuclease-controlled SDR. This phenomenon indicates that polymerase-mediated interference should also receive enough attention in the construction of dynamic DNA nanodevices which work in complex biological media, considering that previous reports only focused on the influence of nuclease on DNA structure.

Inspired by the property of polymerase, we have made use of polymerase-inhibited SDR to establish a keypad lock system (Fig. 4C), whose output signals not only depend on the proper combination but also the correct sequence of input signals, namely password. Since the polymerase only adds nucleotide from 3′-end of DNA strand, we can alternatively inhibit SDR through the selective addition of substrate (i.e., dATP, dTTP, dCTP, and dGTP), leading to 24 kinds of input sequences. The designed sequence of toehold is 5′-CCCCCCGAT-3′, so it requires polymerase to add nucleotide from 3′-end of Q in an order of ATCG, resulting in a complete suppression of SDR. The output signal is defined as “ON” when the SDR is totally inhibited and as “OFF” when SDR is inadequately inhibited. Stimulated by the nucleotide-encoded inputs, the system exhibits different fluorescent responses as shown in Fig. 4D. Obviously, only one correct order of the input signals (ATCG) results in the “ON” state, while all others produce the “OFF” state. Thus, a four-input molecular keypad lock has been successfully mimicked that can stop SDR only when the correct “password” is entered. The system keeps “OFF” at all other combinations to deny the illegal invasion.

**Figure 5:**
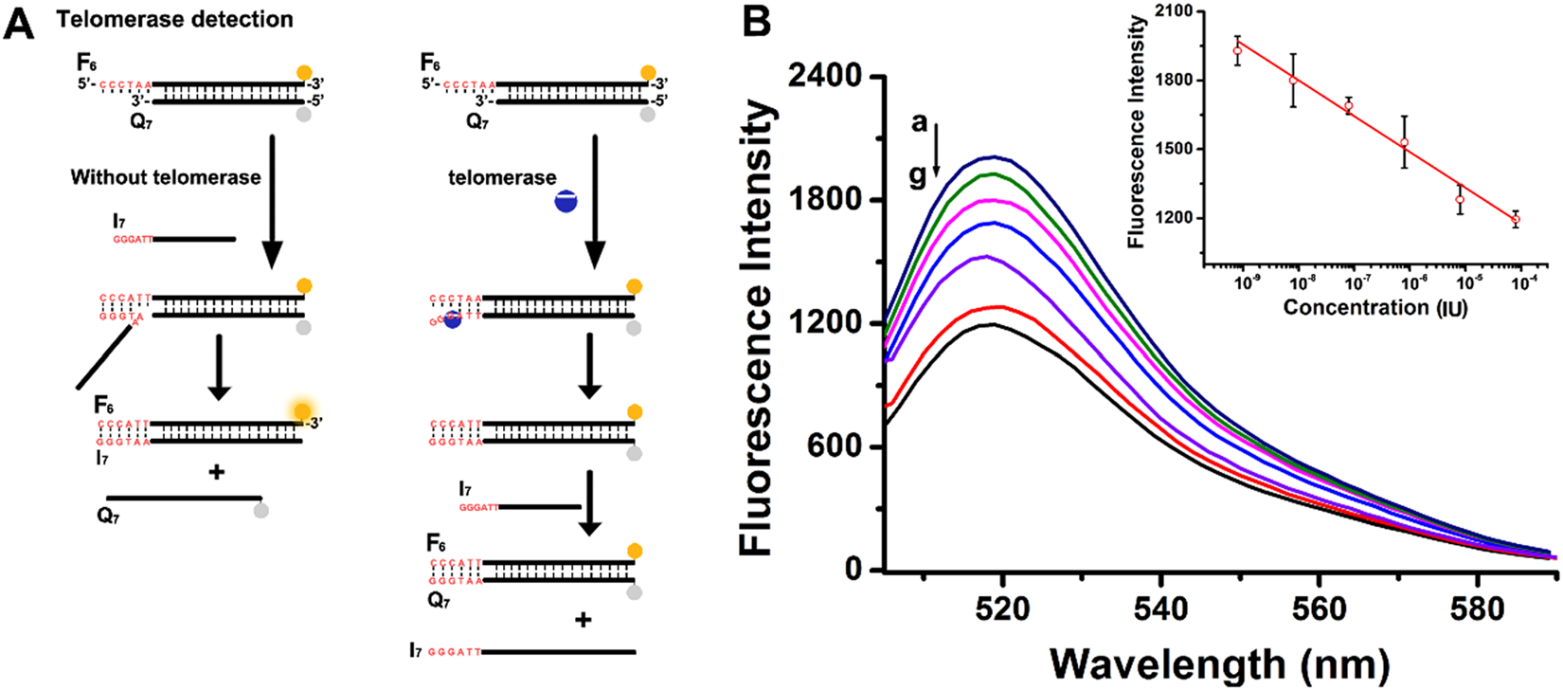
(**A**) General scheme of the telomerase detection based on DNA displacement reaction. (**B**) Fluorescence spectra changes observed upon treatment of the sensing module with variable concentrations of polymerase: (a) 0, (b) 8 × 10^−10^, (c) 8 × 10^−9^, (d) 8 × 10^−8^, (e) 8 × 10^−7^, (f) 8 × 10^−6^, (g) 8 × 10^−5^ IU, [F_6_] = [Q_7_] = [I_7_] = 50 nM.

Also, inspired by the sensitivity of polymerase, we have made use of this inhibition strategy to detect telomerase. Telomerase is a ribonucleoprotein that contains a RNA template in the protein backbone^28^. It is over-expressed in cancer cells, and catalyzes the addition of the telomeric repeats (TTAGGG)_n_ onto the 3′-end of the human chromosomes^29^. As shown in Fig. 5A, the sequence of strand Q_7_ is used as telomerase primer, and the toehold in the strand F_6_ is complementary to the production of telomerase (only one copy). Similar results with polymerase, the elongation of template results in complete blocking of toehold and invalid SDR. Figure 5B depicts the fluorescence spectra generated by the telomerase-treated F_6_Q_7_ that are interacted with invading strand I_7_, in the presence of the dNTP mixture, for a fixed time-interval of 40 min. As the telomerase content increases, the resulting fluorescence is decreased, consistent with the higher level of toehold blocking. Figure 5B, inset, reveals the calibration curve corresponding to the fluorescence intensities generated upon detecting various concentrations of the telomerase. The detection limit for analyzing telomerase corresponded to 8 × 10^−10^ IU. Actually, the potential interference of polymerase can’t be ignored (Figure S2), so we have updated the sequence design by adding a mismatch base (G) in the toehold and employed a novel dNTP mixture without addition of dCTP. As a result, the interference of polymerase can be completely eliminated (Figure S3). All in all, these results demonstrate that SDR can be developed to detect telomerase conveniently and sensitively.

## Conclusion

In summary, we report a series of NAE-based strategies for SDR regulation and offers additional information on how NAEs affect SDR. We have found that (1) Exo III cannot timely stop SDR once the reaction starts, indicating a slower cleavage rate of enzyme compared with DNA displacement; (2) NEase allows the fine regulation of SDR kinetics through adjusting the concentration and recognition site of the enzyme; (3) polymerase should be a unneglected factor that affects nucleic acid system other than widely studied nuclease and can be used to construct biocomputing security system; (4) SDR can be developed to detect nucleic acid-related enzyme. We anticipate that our strategy can also be applied to construct diverse NAE-fueled DNA nanodevices, which may provide a means for biosensing in biological system.

## Methods

### Materials and Reagents

All HPLC-purified DNA oligonucleotides were purchased from Sangon Inc. (Shanghai, China). The DNA sequences and modifications are listed in Table S1. Exonuclease III (Exo III), Nt.BbvCl, *Taq* polymerase and their corresponding reaction buffers were purchased from NEB (Ipswich, USA). Pure telomerase was obtained from the ELISA kit that was purchased from Yibang biotech. Inc. (Suzhou, China). Proteins were diluted with NHS-magnetic bead (diameter = 500 nm) was purchased from Beaver biotech. Inc. (Suzhou, China). Tris(hydroxymethyl)metyl aminomethane (Tris), ethylene diamine tetraacetic acid (EDTA), deoxyadenosine triphosphate (dATP), deoxythymine triphosphate (dTTP), deoxycytosine triphosphate (dCTP), and deoxyguanosine triphosphate (dGTP) and all other chemicals were purchased from Sigma-Aldrich (St. Louis, MO, USA).

The buffers employed in this work were as follows. DNA storage buffer: 20 mM Tris-HCl, 1 mM EDTA (pH 7.4). Exo III reaction buffer: 1 × NEBuffer1. Nt.BbvCl reaction buffer: 1 × CutSmart. *Taq* polymerase reaction buffer: One*Taq* standard reaction buffer. Telomerase reaction buffer: 10 mM phosphate buffered saline (PBS, pH 8.0) with 140 mM KCl, 1.5 mM MgCl_2_, 0.005% tween-20, 200 μM dNTP, and BSA 0.1 mg/ mL. Buffers for both electrochemistry and electrode washing are 10 mM PBS solution with 0.1 M NaCl and 0.05% Tween-20 (pH 7.4). All solutions were prepared with NANOpure H_2_O (>18.0 MΩ) from a Millipore system.

### Fluorescence Measurements

For preparation of **FQ** duplex, strand **F** was mixed with strand **Q** labeled by the 3′- fluorophore and the 5′-quencher at 1:1 ratio in reaction buffer with a final concentration of 50 nM for each strand. The resulting solutions were annealed by heating at 95 °C for 5 min and then slowly cooled down to room temperature over 2 hours. Fluorescence measurements were performed by using an F-7000 fluorescence spectrometry (Hitachi, Japan) at 25 °C. Sample solutions were excited at 494 nm (FAM) and 536 nm (HEX), and the emission signals were recorded with wavelength of 518 nm and 556 nm, respectively. For all time-dependent fluorescence tests, appropriate volumes of DNA stock solutions were added to reaction buffer to achieve 50 nM final concentration with a total volume of 198 μL in the cuvettes. Afterwards, corresponding concentration of enzyme and invading strand **I**, with 1 μL and 1 μL of volume, respectively, were added respectively and mixed quickly within 20 s. In all graphs, time *t* = 0 indicates the time of strand **I** being added to the solutions.

### Keypad lock system

For achieve keypad lock system, NHS-magnetic beads (d = 500 nm) were used as solid support for the DNA strands immobilization. The amino group modified strand **F**_**5**_ was pre-mixed with equivalent strand **Q**_**6**_ (5 μM) and the duplex was tethered onto the surface of NHS-magnetic beads according to the manufacturer instructions. Four deoxynucleotide triphosphates were used as the input signals (A, T, C, G). The beads were exposed to 1 mL of solution composed of 5 U *Taq* polymerase and one of the four deoxynucleotide triphosphates (0.5 mM) for 5 min intervals before being reacted with the next solution. After the reaction with four solutions, the beads were exposed to strand **I**_**6**_ (1 μM) for 5 min intervals and the resulting supernatants were transferred to a cuvette for fluorescent measurements.

### Telomerase detection

For telomerase detection, strand **F**_**6**_ was mixed with strand **Q**_**7**_ at 1:1 ratio in reaction buffer with a final concentration of 50 nM for each strand. The resulting solutions were annealed by heating at 95 °C for 5 min and then slowly cooled down to room temperature over 2 hours. Different concentrations of telomerase were incubated with the duplex for 40 min at 37 °C. Afterward, the resulting solution was introduced into strand I_7_ for 5 min of incubation and was subjected to fluorescent measurements.

## Electronic supplementary material

SUPPLEMENTARY INFO
