# Supplementary material for: Flexible regulation of DNA displacement reaction through nucleic acid-recognition enzyme and its application in keypad lock system and biosensing
Source: Sci Rep. 2017 Aug 30;7:10017. doi: 10.1038/s41598-017-10459-y (PMC5577262; doi:10.1038/s41598-017-10459-y)
Supplement: Supplementary file 1 — SUPPLEMENTARY INFO [file 41598_2017_10459_MOESM1_ESM.docx]

**Supporting Information for**

**Flexible regulation of DNA displacement reaction through nucleic acid-recognition enzyme and its application in keypad lock system and biosensing**

Chao Li^1^, Liu Shi^1^, Yaqin Tao^1^, Xiaoxia Mao^2^, Yang Xiang^1^, Genxi Li^1, 2,^^[[1]](#footnote-1)^*

^1^State Key Laboratory of Pharmaceutical Biotechnology and Collaborative Innovation Center of Chemistry for Life Sciences, Department of Biochemistry, Nanjing University, Nanjing 210093, P. R. China

^2^Center for Molecular Recognition and Biosensing, School of Life Sciences, Shanghai University, Shanghai 200444, P. R. China

**To whom correspondence should be addressed. Tel.: +86-25-83593596, E-mail:* [*genxili@nju.edu.cn*](mailto:genxili@nju.edu.cn)*.*

**Table S1.** DNA oligonucleotide sequences used in this study.

| **Strategy** | **Name** | **Sequence (5’→3’)** |
| --- | --- | --- |
| Exo III-controlled SDR | F_1_ | TCAACATCAGTCTGATAAGCTACT-(Hex)-AAAA |
|  | Q_1_ | (BHQ1)-AGTAGCTTATCAGACTAAAAGATGTTGA |
|  | Q_2_ | (BHQ1)-AGTAGCTTATCAGACTAAAA |
|  | I_1_ | TAGCTTATCAGACTGATGTTGAAAAA |
|  | I_2_ | AGTAGCTTATCAGACTGATGTTGA |
| Nt.BbvCl-inhibited SDR | F_2_ | GCTGAGGAGTTGGCATGAAGT-(Hex) |
|  | F_3_ | CTAGGAGTCGAGTTAGCATGAAGT-(Hex) |
|  | Q_3_ | (BHQ1)- ACTTCATGCCAACT |
|  | Q_4_ | (BHQ1)-ACTTCATGCTAACTCGA |
|  | I_3_ | ACTTCATGCCAACTCCTCAGC |
|  | I_4_ | ACTTCATGCTAACTCGACTCCTAG |
| Polymerase-inhibited SDR | F_4_ | TGGCAGACTGCTCGACGGATT-(FAM) |
|  | Q_5_ | (BHQ1)-AATCCGTCGAGCAG |
|  | I_5_ | AATCCGTCGAGCAGTCTGCCA |
| Keypad lock system | F_5_ | (NH_2_)-CCCCCCGATCCTGCTCGACGGATT-(FAM) |
|  | Q_6_ | (BHQ1)-AATCCGTCGAGCAGG |
|  | I_6_ | AATCCGTCGAGCAGGATCGGGGGG |
| Telomerase detection | F_6_ | CCCATTCCTGCTCGACGGATT-(FAM) |
|  | Q_7_ | (BHQ1)-AATCCGTCGAGCAGG |
|  | I_7_ | AATCCGTCGAGCAGGAATGGG |

Red: toehold sequence, green: space sequence, blue: Nt.BbvCl recognition site.





**Figure S1.** Exo III-mediated fluorescence recovery of F_1_/I_2_ duplex through cleavage of strand I_2_.

**

**

**Figure S2.** The interference of telomerase detection from polymerase. Control: without addition of any enzyme to the telomerase detection system.


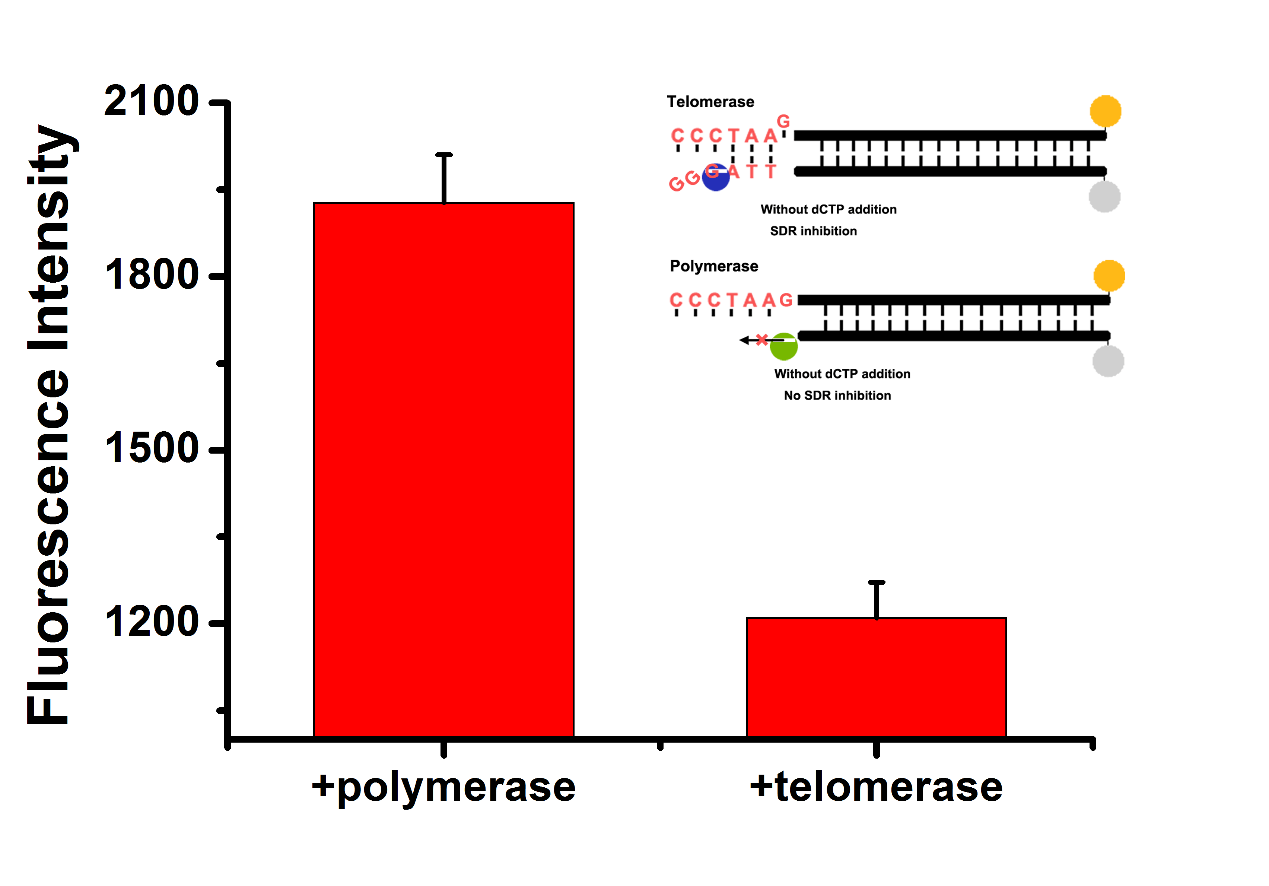


**Figure S3.** Elimination of the interference of telomerase detection though designing the DNA probe and using renewed enzyme substrate (dNTP without containing dCTP).

**Table S2.** Comparative results between the proposed strategy and those already published ones.

| Responsive element | Responsive range or rate | Application | Reference |
| --- | --- | --- | --- |
| DNA and ATP | 0.05 to 50 nM (DNA detection)  0.05 to 500 μM  (ATP detection) | Biosensor and Biocomputing | 1 |
| DNA | 6 × 10^2^- 1.8 × 10^5^ M/s | No report | 2 |
| ATP | 0-1.5 mM | Biosensor | 3 |
| Sr^2+^ | 0-20 mM | Biosensor | 4 |
| Light initiated DNA displacement | 20-200 nM | Biosensor | 5 |
| pH | 1-100 nM | Biosensor | 6 |
| Nucleases | 0-10 units  (Exo III and Nt.BbvCl detection)  1 × 10^-5^-0.1 units  (polymerase detection)  8 × 10^-10^-8 × 10^-5^ IU  (telomerase detection) | Biosensor and Biocomputing | This work |

**References**

1. Guo, Y., Wu, J., & Ju, H. Target-driven DNA association to initiate cyclic assembly of hairpins for biosensing and logic gate operation. *Chem. Sci.* **6**, 4318-4323 (2015).

2. Yang, X., Tang, Y., Traynor, S. M. & Li, F. Regulation of DNA strand displacement using an allosteric DNA toehold. *J. Am. Chem. Soc.* **138**, 14076-14082 (2016).

3. Xing, Y., Yang, Z. & Liu, D. A responsive hidden toehold to enable controllable DNA strand displacement reactions. *Angew. Chem. Int. Ed.* **50**, 11934-11936 (2011).

4. Tang, W. *et al.* DNA tetraplexes-based toehold activation for controllable DNA strand displacement reactions. *J. Am. Chem. Soc.* **135**, 13628-13631 (2013).

5. Huang, F. *et al.* DNA branch migration reactions through photocontrollable toehold formation. *J. Am. Chem. Soc.* **135**, 7967-7973 (2013).

6. Amodio, A. *et al.* Rational design of pH-controlled DNA strand displacement. *J. Am. Chem. Soc.* **136**, 16469-16472 (2014).

1. *Corresponding author: Department of Biochemistry, Nanjing University, Nanjing 210093, China. Fax: +86 25 83592510. E-mail address: genxililab@nju.edu.cn. [↑](#footnote-ref-1)
